# Rapid Whole-Genome Sequencing for Surveillance of *Salmonella enterica* Serovar Enteritidis

**DOI:** 10.3201/eid2008.131399

**Published:** 2014-08

**Authors:** Henk C. den Bakker, Marc W. Allard, Dianna Bopp, Eric W. Brown, John Fontana, Zamin Iqbal, Aristea Kinney, Ronald Limberger, Kimberlee A. Musser, Matthew Shudt, Errol Strain, Martin Wiedmann, William J. Wolfgang

**Affiliations:** Cornell University, Ithaca, New York, USA (H. den Bakker, M. Wiedmann);; US Food and Drug Administration, College Park, Maryland, USA (M.W. Allard, E.W. Brown, E. Strain);; New York State Department of Health/Wadsworth Center, Albany, New York, USA (D. Bopp, R. Limberger, M. Shudt, K.A. Musser, W.J. Wolfgang);; Connecticut Department of Public Health, Rocky Hill, Connecticut, USA (J. Fontana, A. Kinney);; Wellcome Trust Centre for Human Genetics, Oxford, UK (Z. Iqbal)

**Keywords:** Salmonella enterica serovar Enteritidis, bacteria, high-throughput nucleotide sequencing, whole-genome sequencing, pulsed-field gel electrophoresis, infectious disease outbreaks, public health laboratory surveillance

## Abstract

For *Salmonella enterica* serovar Enteritidis, 85% of isolates can be classified into 5 pulsed-field gel electrophoresis (PFGE) types. However, PFGE has limited discriminatory power for outbreak detection. Although whole-genome sequencing has been found to improve discrimination of outbreak clusters, whether this procedure can be used in real-time in a public health laboratory is not known. Therefore, we conducted a retrospective and prospective analysis. The retrospective study investigated isolates from 1 confirmed outbreak. Additional cases could be attributed to the outbreak strain on the basis of whole-genome data. The prospective study included 58 isolates obtained in 2012, including isolates from 1 epidemiologically defined outbreak. Whole-genome sequencing identified additional isolates that could be attributed to the outbreak, but which differed from the outbreak-associated PFGE type. Additional putative outbreak clusters were detected in the retrospective and prospective analyses. This study demonstrates the practicality of implementing this approach for outbreak surveillance in a state public health laboratory.

For genetically monomorphic bacteria, current typing methods often prove inadequate for outbreak detection, trace back, and identification of transmission routes. Some of these bacteria, such as *Salmonella enterica* serovar Enteritidis, *S*. *enterica* serovar Montevideo, *Staphylococcus aureus*, *Clostridium difficile*, *Klebsiella pneumoniae*, and *Mycobacterium tuberculosis*, cause diseases that have major public health effects. For these pathogens, retrospective studies have unambiguously demonstrated that phylogenetic analysis based on whole-genome–derived single nucleotide polymorphisms (SNPs) improves cluster resolution and would be an invaluable tool in epidemiologic investigations (*1*–*8*). We refer to this approach as whole-genome cluster analysis.

Introduction of small, affordable, and rapid benchtop whole-genome sequencers, such as the Illumina MiSeq (Illumina, San Diego, CA, USA) MiSeq and the Ion Torrent PGM (Life Technologies, Carlsbad, CA, USA), has made it possible for clinical and public health laboratories to contemplate adding genome sequencing as a rapid typing tool. Eyre et al. (*9*) showed the utility of Illumina MiSeq in the detection of nosocomial outbreaks of *S*. *aureus* and *C*. *difficile* infections. Although Eyre et al. (*9*) showed the utility of this approach in improving typing of these monomorphic pathogens, the utility of these sequencers in a larger public health setting, in which capacity and turn-around times are critical parameters, has not been demonstrated.

The standard typing method for *Salmonella* species, which is used by PulseNet laboratories, is pulsed-field gel electrophoresis (PFGE) (*10*). However, PFGE has limited discriminatory power for *S. enterica* serovar Enteritidis strains and clusters. At the New York State Department of Health (NYSDOH) Wadsworth Laboratories (Albany, NY, USA), ≈50% of the 350–500 *S. enterica* serovar Enteritidis isolates received each year are PFGE type JEGX01.0004. Multilocus variable-number tandem-repeat analysis (MLVA) of these isolates improves discrimination of disease clusters for this pathogen, but even this tool assigns 30% of isolates to a single MLVA type. Because genomic homogeneity of *S. enterica* serovar Enteritidis is also observed on a national and international level (*11*), a clear need exists for improved typing methods for *S. enterica* serovar Enteritidis in the public health laboratory.

To determine if whole-genome cluster analysis can improve subtype discrimination and cluster detection in the public health laboratory, we sequenced 93 *S. enterica* serovar Enteritidis isolates received during routine surveillance activities at the NYSDOH by using the Ion Torrent PGM located in the core sequencing facility. The sequence data were used to create SNP-based phylogenetic trees. This study consisted of 2 parts. First, we conducted a retrospective analysis that focused on an epidemiologically defined outbreak of *S. enterica* serovar Enteritidis JEGX01.0004 within a long-term care facility (LTCF). Second, we conducted a prospective study in which nearly all *S. enterica* serovar Enteritidis PFGE patterns JEGX01.0004 and JEGX01.0021 were sequenced during a 4-month period during the summer of 2012. In addition, we retrospectively sequenced JEGX01.0009 *S. enterica* serovar Enteritidis isolates that had been associated with contaminated ground beef early in the summer of 2012.

The retrospective part of the study serves as a proof of principle and clearly demonstrates increased resolution of whole-genome cluster analysis for typing of common PFGE pattern types and subsequent outbreak detection. In the prospective study, we show the feasibility of detecting outbreaks in near real time, as well as improved resolution, of the method that enables detection of numerous potential outbreak clusters that would likely go undetected by PFGE.

## Materials and Methods

Ninety-three *S. enterica* serovar Enteritidis isolates received at the Wadsworth Center were selected from our routine surveillance (Technical Appendix Table). Serotype, PFGE PulseNet pattern and NYS MLVA designation were determined by using standard methods (*10*,*12*–*14*) before sequencing. Retrospective study isolates had been collected during August 10, 2010–October 22, 2011. For this period, we sequenced all isolates with the outbreak–associated PFGE pattern JEGX01.0004 and NYS-MLVA pattern W (JEGX01.0004/NYS-W) (n = 28), selected isolates with the most common pattern JEGX01.0004/NYS-B (n = 6), and 1 isolate with pattern JEGX01.0004/NYS-AE, which was initially believed to be part of the outbreak. These isolates represent 5% of all *S. enterica* serovar Enteritidis isolates and 10% of all pattern JEGX01.0004 isolates received by the Wadsworth Center during this period.

Prospective study isolates were obtained during April 17, 2012–August 16, 2012. Isolates sequenced from this period included all JEGX01.0004/NYS-B (n = 22) except 2, all JEGX01.0004/NYS-W except 1 (n = 3), all JEGX01.0021/NYS-B (n = 22), selected JEGX01.0009/NYS-CR (n = 8), and 1 isolate each of JEGX01.0843/NYS-CR, JEGX01.0968/NYS-CR, and JEGX01.0034/NYS-B. These isolates represented 9% of all *Salmonella* species isolates received by our laboratory over the 4-month period. In addition, sequence data from earlier studies of *S. enterica* serovar Enteritidis (*11*,*15*) were included in the data analysis.

Enzymatic shearing of genomic DNA samples and generation of barcoded libraries were carried out by using the Ion Xpress Plus Fragment Library Kit and Ion Xpress Barcode Adapters Kit (Life Technologies). Libraries were size-selected by using a 2-step AMPure XP isolation method that was optimized for use in Ion 200 bp Template kits (*16*). Templates were prepared by using the Ion OneTouch 200 Template Kit version 2 and the Ion OneTouch system. Sequencing was conducted by using the Ion PGM with a 316 chip and the Ion PGM 200 Sequencing Kit. The Torrent Suite versions 2.0.1–2.2 (https://olex-secure.openlogic.com/packages/ts-iontorrent/2.2) was used for base calling. Isolates were sequenced with an average coverage of 16–135 times (median 53 times).

Two SNP detection methods were used in this study: a traditional reference-based method that used VarScan (*17*) for SNP detection, and a de novo genomic variant detection method as implemented in Cortex Variation Assembler (*18*). The traditional reference-based pipeline relied on BWA 0.6.1 (*19*) to map reads against a reference genome, and VarScan was used for SNP detection. Only SNPs that were in agreement with the following parameters were used in the analysis: minimal coverage of 8, minimal variant coverage of 8, minimum variant frequency of 90%, and a p value ≤0.01. A consensus sequence was created by using vcftools (http://vcftools.sourceforge.net/), and sites with coverage <8 were hard-masked in the consensus sequence.

The consensus sequences were used as input for BRATNextGen (*20*), a homologous recombination detection software. Recombinogenic regions detected by BRATNextGen and rRNA-encoding regions were excluded from further analyses. The publicly available genome sequence of *S. enterica* serovar Enteritidis P125109 (GenBank accession no. NC_011294) and a de novo–assembled draft genome of *S. enterica* serovar Enteritidis 10_34587 (GenBank accession no, AWOI00000000) were used as references in the analysis. The de novo assembly of *S. enterica* serovar Enteritidis 10_34587 was created by using MIRA version 3.2.1 (*21*).

Cortex_var version v1.0.5.14 (http://cortexassembler.sourceforge.net/index_cortex_var.html) was used for de novo variant detection. The run calls script was used to call variants by using the independent work flow (*18*) by using the bubble caller. Read filter parameters were adjusted so that the maximum possible read length could be used without use of an excessive amount of random access memory. For the Ion Torrent, reads used in this study bases with a Phred score <15 were clipped and reads were split when homopolymers longer than 5 bp were encountered. SNPs and indels were used for further analysis if they passed the Cortex population filter/site classifier by using default parameters.

Population-level phylogenetic analysis was performed only on variable sites. Only sites that were correctly called in ≥95% of the isolates were included in the analysis. Maximum-likelihood–based phylogenetic inference and nucleotide substitution model selection were performed in MEGA 5.1 (*22*). A bootstrap analysis based on 150 bootstrap replicates was performed to assess robustness of individual clades.

## Results

### Identification of SNPs

Reference mapping against the publicly available genome sequence of *S. enterica* serovar Enteritidis P125109 yielded 1,240 SNPs among the 93 newly sequenced isolates from New York, and the de novo pipeline yielded 903 SNPs for the same dataset; 714 SNPs were called by both pipelines. Phylogenetic analysis showed the same population structure based on the reference-derived dataset, the de novo–derived dataset, and a dataset consisting of SNPs called by both pipelines. Results of the reference-based pipeline are reported in the remainder of this study. Addition of 41 previously sequenced isolates (*11*,*15*) to the reference mapping–based pipeline increased the number of SNPs to 4,510. After exclusion of regions that were putatively affected by homologous recombination and sites that were called in <95% of the isolates, 2,031 SNPs were used for further analysis.

### Retrospective Study

To determine if whole-genome cluster analysis could improve the resolution of outbreak clusters for *S. enterica* serovar Enteritidis, we selected a retrospective cohort from an epidemiologically defined outbreak that occurred in Connecticut and New York during September 1, 2010–September 30, 2010. This outbreak was associated with an LTCF. The study cohort (isolates obtained during August 10, 2010–October 22, 2011) contained 7 JEGX01.0004/NYS-W (combined PulseNet PFGE type and NYS-MLVA type) isolates that were epidemiologically linked to the outbreak and 21 JEGX01.0004/NYS-W, 6 JEGX01.0004/NYS-B, and 1 JEGX01.0004/NYS-AE that were considered to be from sporadic outbreaks (Table).

**Table:** Retrospective cohort of *Salmonella enterica* serovar Enteritidis isolates analyzed by whole-genome cluster analysis *

ID no.	Collection date	State	PFGE-MLVA combined†	Cluster detected by epidemiology‡	Cluster detected by WGCA‡
10_28670	2010 Aug 8	NY	JEGX01.0004-B	–	–
10_29153	2010 Aug 10	NY	JEGX01.0004-W	–	–
10_29949	2010 Aug 16	NY	JEGX01.0004-B	–	–
10_30147	2010 Aug 22	NY	JEGX01.0004-W	–	Cluster B
10_31528	2010 Aug 26	NY	JEGX01.0004-W	–	–
10_33213	2010 Sep 10	NY	JEGX01.0004-W	–	LTCF
10_33369	2010 Sep 10	NY	JEGX01.0004-W	–	LTCF
10_33371	2010 Sep 11	NY	JEGX01.0004-W	–	LTCF
10_35179	2010 Sep 12	CT	JEGX01.0004-W	LTCF	LTCF
10_35180	2010 Sep 12	NY	JEGX01.0004-W	LTCF	LTCF
10_35182	2010 Sep 12	NY	JEGX01.0004-W	LTCF	LTCF
10_35178	2010 Sep 13	NY	JEGX01.0004-W	LTCF	LTCF
10_35181	2010 Sep 13	NY	JEGX01.0004-W	LTCF	LTCF
10_34601	2010 Sep 13	NY	JEGX01.0004-W	–	LTCF
10_34213	2010 Sep 13	NY	JEGX01.0004-B	–	–
10_33603	2010 Sep 14	NY	JEGX01.0004-B	–	–
10_34599	2010 Sep 15	NY	JEGX01.0004-W	–	Cluster A
10_35183	2010 Sep 16	CT	JEGX01.0004-W	LTCF	LTCF
10_35184	2010 Sep 16	NY	JEGX01.0004-AE	–	–
10_36119	2010 Sep 17	NY	JEGX01.0004-W	LTCF	LTCF
10_34587	2010 Sep 20	NY	JEGX01.0004-W	–	LTCF
10_35417	2010 Sep 22	NY	JEGX01.0004-W	–	LTCF
10_36319	2010 Sep 28	NY	JEGX01.0004-W	–	LTCF
10_37723	2010 Oct 4	NY	JEGX01.0004-B	–	–
10_36979	2010 Oct 8	NY	JEGX01.0004-W	–	LTCF
10_39087	2010 Oct 27	NY	JEGX01.0004-B	–	–
10_38792	2010 Oct 29	NY	JEGX01.0004-W	–	LTCF
11_03844	2011 Feb 1	NY	JEGX01.0004-W	–	Cluster B
11_06235	2011 Feb 21	NY	JEGX01.0004-W	–	Cluster A
11_21079	2011 Jul 13	NY	JEGX01.0004-W	–	Cluster A
11_22186	2011 Jul 22	NY	JEGX01.0004-W	–	Cluster B
11_27690	2011 Sep 6	NY	JEGX01.0004-W	–	Cluster B
11_31312	2011 Oct 5	NY	JEGX01.0004-W	–	Cluster B
11_30508	2011 Oct 9	NY	JEGX01.0004-W	–	Cluster B
11_32014	2011 Oct 22	NY	JEGX01.0004-W	–	Cluster B

**Figure 1:**
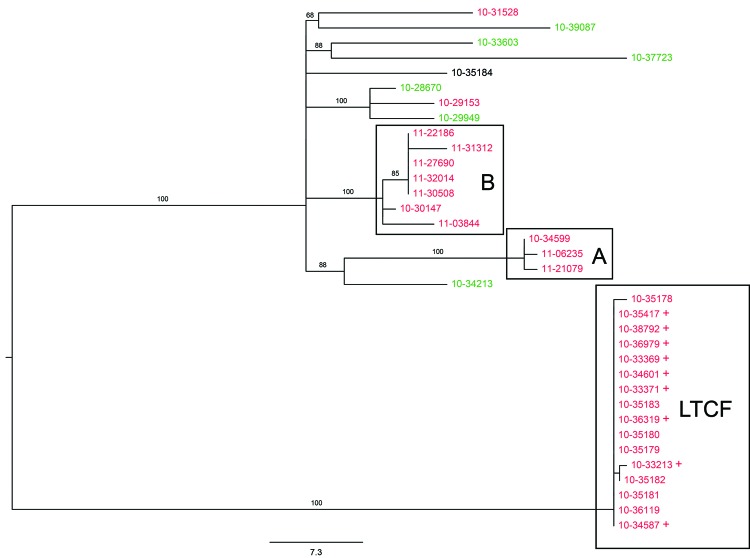
Maximum-likelihood tree of population structure of *Salmonella enterica* serovar Enteritidis isolates obtained in New York and neighboring states, USA. The tree was inferred by using a general time-reversible model with a gamma distribution and was inferred to be the best fit model by the maximum-likelihood method implemented in MEGA 5.1 (*22*). Values on branches are bootstrap values based on 150 bootstrap replicates. Note the well supported and distant cluster associated with the long-term care facility (LTCF), as well as additional clusters A and B. Labels of isolates are colored according to their New York State Department of Health Wadsworth Laboratories multilocus variable-number tandem-repeat analysis (MLVA) subtype designation. Green, MLVA subtype B; red, MLVA subtype W; black, MLVA subtype AE. + indicates isolates in the LTCF cluster that were detected only by whole-genome analysis and were not detected epidemiologically. Scale bar indicates single-nucleotide polymorphisms per site.

*ID, identification; PFGE, pulsed-field gel electrophoresis; MLVA, multilocus variable-number tandem-repeat analysis; WGCA, whole-genome cluster analysis; NY, New York; –, sporadic outbreaks; LTCF, isolates assigned to long-term care facility outbreak; CT, Connecticut. †The letter designating the New York MLVA type follows PulseNet PFGE pattern designation. ‡Cluster designations are indicated by boxes in Figure 1.

Maximum-likelihood analysis of the SNP matrix placed the epidemiologically defined outbreak isolates in a well-supported clade with an average pairwise SNP difference of <1.0 (Figure 1). The clade is 78 SNPs distant from the nearest neighbor in the cohort. Whole-genome cluster analysis identified 9 additional isolates as part of this outbreak cluster (Table; Figure 1). These additional isolates were obtained during the time of the outbreak in the same regions of New York and Connecticut and showed pattern JEGX01.0004/NYS-W, but were not epidemiologically linked to the LTCF at the time of the outbreak (Table). No attempt was made to link these isolates to the LTCF outbreak. On the basis of whole-genome cluster analysis, 12 JEGX01.0004/NYS-W, 6 JEGX01.0004/NYS-B, and 1 JEGX01.0004/NYS-AE were unambiguously excluded from the outbreak. Excluded isolates were characterized by an MLVA type other than NYS-W or were obtained at distant sites or at times outside the outbreak period. Among these excluded isolates, we detected 2 additional clusters (clusters A and B) not associated with any known outbreak (Table; Figure 1).

### Prospective Study

To further evaluate the application of whole-genome cluster analysis to *S. enterica* serovar Enteritidis typing and cluster detection, we sequenced isolates with 2 of the most common combined PFGE/MLVA patterns (JEGX01.0004/NYS-B and JEGX01.0021/ NYS-B) as they were obtained during April 17, 2012–August 16, 2012. All JEGX01.0004/NYS-W isolates, the type associated with the LTCF outbreak, were sequenced to determine if this clone persisted. In addition, we conducted a retrospective analysis of isolates from another outbreak (Technical Appendix Table) to test the performance of whole-genome cluster analysis in a second bona fide outbreak. Trees were constructed in an ad hoc manner as data were acquired, which was not real time because of slow turnaround times.

**Figure 2:**
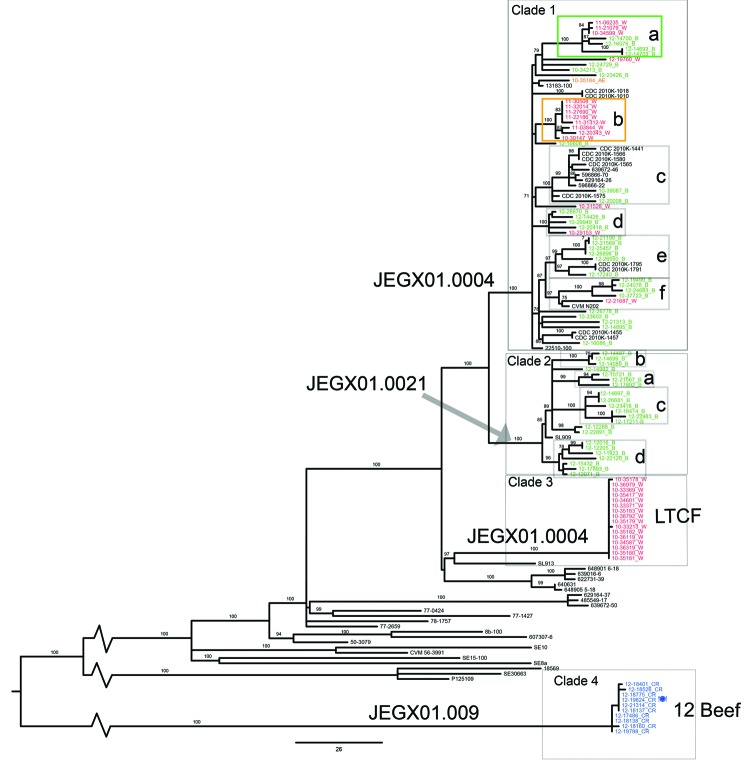
Maximum-likelihood tree of population structure of *Salmonella enterica* serovar Enteritidis isolates obtained in New York and neighboring states, USA. The tree was inferred by using a general time-reversible model with a gamma distribution, which was inferred to be the best fit model by the maximum-likelihood method implemented in MEGA 5.1 (*22*). Values on branches are bootstrap values based on 150 bootstrap replicates. Pulsed-field gel electrophoresis (PFGE) types are indicated on branches. Labels of isolates are colored according to their New York State Department of Health Wadsworth Laboratories multilocus variable-number tandem-repeat subtype designation. Green, MLVA subtype B; red, MLVA subtype W; orange, MLVA subtype AE; blue, MLVA subtype CR; black, MLVA subtype data missing and isolates from Allard et al. (*11*). Rectangles indicate well-supported clusters of at least 3 isolates, letters within the rectangles correspond to the cluster designation in the Table. LTCF, long-term care facility. Scale bar indicates single-nucleotide polymorphisms per site.

Whole-genome sequence data for these 58 isolates, as well as for 41 isolates that were sequenced as part of a large study of *S. enterica* serovar Enteritidis infections that were associated with eggs (*11*,*15*), were combined with the whole-genome sequence data from the retrospective study described above. Four well-supported (bootstrap values 100) clades were apparent (Figure 2). Clade 1 contained 62 isolates with PulseNet PFGE type JEGX01.0004 and 1 isolate with PFGE type JEGX01.0034. Clade 2 contained 23 isolates with PFGE type JEGX01.0021. Clade 3 contained 16 isolates associated with the 2010 LTCF outbreak described above. Clade 4 contained 10 isolates associated with a 2012 outbreak linked to consumption of contaminated ground beef (http://www.cdc.gov/salmonella/enteritidis-07-12/) and represented PFGE types JEGX01.0009 (n = 8), JEGX01.0843 (n = 1), or JEGX01.0968 (n = 1). Clades 1–3 all belong to *S. enterica* serovar Enteritidis lineage V, a clade that is the prevalent lineage in the United States and is predominately associated with poultry products, such as shelled eggs and broilers (K. Deng et al., unpub. data). Clades 1 and 2 also contained isolates from clade C2 associated with the shelled egg outbreak in 2010 (*11*,*15*). Clade 4 belongs to *S. enterica* serovar Enteritidis lineage II, which is rare among *S. enterica* serovar Enteritidis isolates from the United States, but has been isolated from mammalian hosts (K. Deng et al., unpub. data).

Clades 3 and 4 are composed of isolates from epidemiologically defined outbreaks that are divergent from other *S. enterica* serovar Enteritidis isolates in this study. Within-clade variability is limited for both clades (clade 3: 0–2 pairwise SNP differences, average pairwise SNP difference <1 SNP; clade 4: 0–3 pairwise SNP differences, average pairwise SNP difference 1.2 SNP), which, together with the short time span from the first isolate to the last isolate, points toward a point source outbreak.

Clade 4 consists of 10 isolates (9 from humans and 1 from contaminated hamburger [12_19824]) from a multistate outbreak in 2012 associated with beef. A total of 46 cases in 9 states were associated with this outbreak (http://www.cdc.gov/salmonella/enteritidis-07-12/). This outbreak was unusual because it was associated with contaminated beef, and *S. enterica* serovar Enteritidis is more commonly associated with poultry products (*23*). Eight of the isolates sequenced were PFGE type JEGX01.0009, and 2 isolates (12_18137 and 12_21314) were PFGE types JEGX01.0968 and JEGX01.0843, respectively (these differ by only 1–2 bands from JEGX01.0009).

Isolates with PFGE type JEGX01.0968 or JEGX01.0843 have the same SNP profile as 2 PFGE type JEGX01.0009 isolates (12_18775 and 12_19824), which suggests that the difference in 3 PFGE types is not associated with differences in the genomic backbone of these isolates. De novo assembly of sequence data for isolates 12_18137 and 12_21314 by using MIRA version 3.2.1 corroborated this finding and showed that each isolate contains ≥1 large plasmids. To assess the distribution of these plasmids among the isolates from the outbreak, we used Cortex_var for a plasmid presence/absence analysis. This analysis showed that these plasmids are absent from all other isolates in this outbreak.

Most isolates obtained by NYSDOH and sequenced in this study belonged to clades 1 and 2. Pairwise SNP differences within the clades are similar: 0–53 pairwise SNP differences (average 29.5) between isolates in clade 1, and 0–42 pairwise SNP differences (average 25.1) between isolates in clade 2. Indicative of a highly structured population, clades 1 and 2 can be further subdivided into 6 and 5 well-supported (bootstrap value >97%) subclades, respectively (Figure 2). These subclades most likely represent strains that persist in the environment (i.e., in poultry) and consequently caused multiple human cases. Evidence for persistence is particularly strong for clade 1, in which 5 of 6 clusters contain isolates obtained during the summer or fall of 2010–summer of 2012. Further research and epidemiologic data are needed to determine if these strains are widely distributed or represent exposure to a specific source.

To assess the distribution of plasmids and prophages, we queried de novo assemblies of representative isolates from each clade and each PFGE type by using Cortex_var. This analysis showed that the *S. enterica* serovar Enteritidis virulence plasmid pSLA5 (GenBank accession no. NC_019002.1) was present in all isolates sequenced, with the exception of 12_23426 from clade 1. This analysis also confirmed the presence of the unique plasmids found by de novo assembly for isolates 12_18137 and 12_21314 described above. In clades 1, 2, and 3, prophage distribution is highly conserved, and all genomes sequenced in this study contained an ELPhiS-like (*24*) prophage. Although the ELPhis-like prophage is absent from all clade 4 isolates, these isolates contain a unique prophage region of ≈49 kb, which was not found in other clades studied here or in currently published *Salmonella* genomes.

## Discussion

In this study, we demonstrated that whole-genome cluster analysis of *S. enterica* serovar Enteritidis results in vastly improved detection of clusters of common PFGE types and outbreak resolution than PFGE, the current standard. Analysis of a retrospective dataset showed that all isolates associated with an LTCF outbreak in 2010 belonged to a well-supported clade with an average <1.0 SNP distance between all the isolates in the clade. Furthermore, this clade is 78 SNPs distant from the nearest neighboring sporadic isolates. Additional clinical isolates obtained during the time of the outbreak from patients in surrounding communities not previously associated with the outbreak also belonged to the clade, which expanded the number of possible outbreak cases from 7 to 16. Identification of these additional 9 matching isolates suggests a common contaminated source outside the LTCF. Knowledge of these cases at the time of the outbreak might have improved the chances of finding the outbreak source, which was never identified. Furthermore, whole-genome cluster analysis showed that the LTCF outbreak belonged to the same monophyletic lineage as isolates in 2 clades associated with the 2010 shelled egg outbreak, suggesting that shelled eggs are a common source of infection (*11*,*15*). For the LTCF outbreak, MLVA data showed concordance with whole-genome sequencing data. In contrast, PFGE analysis of all isolates (i.e., from the LTCF and the shelled eggs outbreak) resulted in a single type (JEGX01.0004), which yielded no useful molecular clustering information.

When we combined retrospective and prospective datasets, no additional isolates clustered with those from the LTCF outbreak (these datasets included 3 JEGX01.0004/NYS-W isolates). However, several other clusters were detected. One well-supported and distant cluster associated with an outbreak linked to contaminated beef contains PFGE patterns JEGX 01.009, JEGX01.0968, and JEGX01.0843. These PFGE types are rarely seen in the United States. Other smaller well-supported clusters were observed that contained isolates obtained during a 2.5-year period, which suggested persistence of point sources in the environment.

During the outbreak associated with contaminated beef, 2 isolates (12_18137 and 12_21314), which have distinct PFGE types (JEGX 01.0968 and JEGX 01.0843, respectively), had not been included in the outbreak. However, whole-genome cluster analysis placed these isolates in the outbreak cluster. De novo assembly of the sequences of these 2 strains showed the presence of plasmids that are not found in other isolates in this clade. This observation is consistent with observations of Zhou et al. (*25*), who found that differences in PFGE types in *S.* *enterica* serovar Agona could be attributed mainly to differences in the content of mobile elements (e.g., prophages and plasmids), and not to SNP-related differences in the genomic backbone. Similar to our observations, Gilmour et al. (*26*) also linked *Listeria monocytogenes* isolates with PFGE types that differed by <3 bands to an outbreak, on the basis of whole-genome sequencing data, which indicated that PFGE pattern diversification was caused by mobile elements.

Other retrospective studies of *S. aureus*, *K. pneumoniae*, *C. difficile*, and *M. tuberculosis* have also demonstrated improved resolution of whole-genome cluster analysis (*2*–*8*,*15*). Whether this approach can be translated to the public health laboratory setting is still unclear. These laboratories currently support the bulk of outbreak investigations through various formal and informal networks. Interpretation of whole-genome sequencing data are relatively straightforward and no more challenging than the interpretation of PFGE or MLVA data. During our prospective study, we were able to sequence 12 isolates at the NYSDOH and analyze the output at the Cornell Food Safety Laboratories within 8 days. Recently, our throughput has increased to 32 isolates in the same period, and analysis can be conducted in house. At this rate, all *Salmonella* isolates received for surveillance can be sequenced in a timeframe that is useful to epidemiologists. In addition, once whole-genome cluster analysis is fully implemented for all *Salmonella* isolates, serovar (*27*) and multilocus sequence typing (*28*) information could be inferred solely from the genome data to link sequenced isolates to historical data, which would shorten the turnaround time by 2 days. Thus, it is reasonable to expect that public health laboratories can serve as centers for these new technologies and will be able to detect clusters in a meaningful time frame.

For national surveillance, whole-genome cluster analysis could use public health laboratories and standardized protocols and procedures to analyze data locally and upload raw sequence data to centralized sites for analysis. This approach builds upon the PulseNet model (*10*) and would enable a rapid local response and centralized control. One model for centralized data is being tested in a collaboration between the US Food and Drug Administration, the National Center for Biotechnology Information, and selected state public health laboratories (http://www.fda.gov/Food/FoodScienceResearch/WholeGenomeSequencingProgramWGS/default.htm). Surveillance laboratories upload raw sequence reads that are processed and added to a single tree that harbors all sequenced *S. enterica* isolates and associated metadata (date of isolation, isolation source, location, unique identifier). As clusters appear, they would be reported to the surveillance laboratories, which would communicate the information to epidemiologists. The uploading, analysis, and reporting could be highly automated.

Many challenges need to be addressed before a whole-genome sequence–based surveillance system can be implemented. In addition to standardization of protocols and analyses, several questions still need to be resolved. What constitutes an epidemiologically meaningful phylogenetic cluster? Do circulating persistent clones confound this analysis? How will this information be reported to epidemiologists? Pilot studies at the Centers for Disease Control and Prevention (Atlanta, GA, USA) and NYSDOH to implement real-time whole-genome–based surveillance for *L. monocytogenes* and *S. enterica* serovar Enteritidis, respectively, will begin to address these questions.

Improving surveillance and tracking of pathogens is a high priority goal for federal and state agencies charged with protecting public health. Affordable and rapid next-generation sequencing technologies and associated bioinformatics will be potent tools in achieving these goals. This study demonstrates the practical feasibility and benefits of deploying these technologies in public health laboratories.

Technical Appendix Isolates of *Salmonella enterica* serovar Enteritidis tested by using rapid whole-genome sequencing.
