# Supplementary material for: Rapid Whole-Genome Sequencing for Surveillance of Salmonella enterica Serovar Enteritidis
Source: Emerg Infect Dis. 2014 Aug;20(8):1306–14. doi: 10.3201/eid2008.131399 (PMC4111163; doi:10.3201/eid2008.131399)
Supplement: Technical Appendix — Isolates of Salmonella enterica serovar Enteritidis tested by using rapid whole-genome sequencing. [file 13-1399-Techapp-s1.pdf]

Article DOI: http://dx.doi.org/10.3201/eid2008.131399

Rapid Whole-Genome Sequencing of *Salmonella enterica* Serovar Enteritidis

Technical Appendix

Technical Appendix Table. Isolates of *Salmonella enterica* serovar Enteritidis tested by using rapid whole-genome sequencing*

Isolate tree label	Collection location	Isolation source	Collection date	PFGE pattern: first enzyme; second enzyme	NY MLVA	Bioproject	SRA accession no.	Clade/subclade†	Specific outbreak	Reference
12_21190	USA: NY	Stool	2012 Jul 5	JEGX01.0004	B	178162	SRR651173	1/e	Sporadic	Current study
12_21569	USA: NY	Stool	2012 Jul 4	JEGX01.0004	B	178162	SRR651176	1/e	Sporadic	Current study
12_25457	USA: NY	Stool	2012 Aug 5	JEGX01.0004	B	178162	SRR652081	1/e	Sporadic	Current study
12_26898	USA: NY	Stool	2012 Aug 16	JEGX01.0004	B	178162	SRR652085	1/e	Sporadic	Current study
12_17240	USA: NY	Stool	2012 May 26	JEGX01.0004	B	178162	SRR651157	1/e	Sporadic	Current study
CDC 2010K-1795	USA: TN	Clinical	2010 Jun 15	JEGX01.0004_JEGA26.0002	NA	52519	SRR518752	1/e	Sporadic	(*1*)
CDC 2010K-1791	USA: TN	Clinical	2010 Jun 25	JEGX01.0004_JEGA26.0002	NA	52517	SRR518839	1/e	Sporadic	(*1*)
12_26550	USA: NY	Stool	2012 Aug 1	JEGX01.0004	B	178162	SRR652082	1/e	Sporadic	Current study
12_19490	USA: NY	Stool	2012 Jun 23	JEGX01.0004	B	178162	SRR651165	1/f	Sporadic	Current study
12_24683	USA: NY	Stool	2012 Aug 1	JEGX01.0004	B	178162	SRR652079	1/f	Sporadic	Current study
12_24078	USA: NY	Stool	2012 Jul 28	JEGX01.0004	B	178162	SRR652078	1/f	Sporadic	Current study
10_37723	USA: NY	Stool	2010 Oct 4	JEGX01.0004	B	178162	SRR618458	1/f	Sporadic	Current study
12_21687	USA: NY	Stool	2012 Jul 7	JEGX01.0004	W	178162	SRR651177	1/f	Sporadic	Current study
CVM N202	USA	Chicken	2009 Jun 27	JEGX01.0004_JEGA26.0002	NA	59989	SRR518828	1/f	Sporadic	(*1*)
CDC 2010K-1580	USA: MN	Clinical	2010 Jul 10	JEGX01.0004_JEGA26.0002	NA	51999	SRR518748	1/c	Sporadic	(*1*)
CDC 2010K-1441	USA: CA	Clinical	2010 Jun 3	JEGX01.0004_JEGA26.0002	NA	52003	SRR518771	1/c	Sporadic	(*1*)
CDC 2010K-1566	USA: MN	Clinical	2010 May 27	JEGX01.0004_JEGA26.0002	NA	51997	SRR518830	1/c	Sporadic	(*1*)
596866–70	USA: IA	Environmental swab	2010 Aug 31	JEGX01.0004; JEGA26.0002	NA	59535	SRR518755	1/c	Sporadic	(*2*)
629164–26	USA: IA	Environmental swab	2010 Aug 30	JEGX01.0034; JEGA26.0002	NA	59537	SRR518756	1/c	Sporadic	(*2*)
CDC 2010K-1565	USA: MN	Clinical	2010 May 30	JEGX01.0004_JEGA26.0002	NA	52501	SRR518766	1/c	Sporadic	(*1*)
639672–46	USA: IA	Environmental swab	2010 Aug 31	JEGX01.0004; JEGA26.0002	NA	59541	SRR518770	1/c	Sporadic	(*2*)
596866–22	USA: IA	Environmental swab	2010 Aug 31	JEGX01.0004; JEGA26.0002	NA	59533	SRR518816, SRR518817	1/c	Sporadic	(*2*)
CDC 2010K-1575	USA: MN	Clinical	2010 Jul 3	JEGX01.0004_JEGA26.0002	NA	52511	SRR518831	1/c	Sporadic	(*1*)
10_39087	USA: NY	Stool	2010 Oct 27	JEGX01.0004	B	178162	SRR618462	1/c	Sporadic	Current study
12_20008	USA: NY	Stool	2012 Jun 25	JEGX01.0004	B	178162	SRR651169	1/c	Sporadic	Current study
10_28670	USA: NY	Blood	2010 Aug 8	JEGX01.0004	B	178162	SRR618448	1/d	Sporadic	Current study
12_20418	USA: NY	Stool	2012 Jun 26	JEGX01.0004	B	178162	SRR651171	1/d	Sporadic	Current study
10_29153	USA: NY	Stool	2010 Aug 10	JEGX01.0004	W	178162	SRR610557	1/d	Sporadic	Current study
10_29949	USA: NY	Stool	2010 Aug 16	JEGX01.0004	B	178162	SRR618449	1/d	Sporadic	Current study
12_14426	USA: NY	Stool	2012 May 9	JEGX01.0004	B	178162	SRR651129	1/d	Sporadic	Current study

Isolate tree label	Collection location	Isolation source	Collection date	PFGE pattern: first enzyme; second enzyme	NY MLVA	Bioproject	SRA accession no.	Clade/subclade†	Specific outbreak	Reference
11_27690	USA: NY	Stool	2011 Sep 6	JEGX01.0004	W	178162	SRR618470	1/b	Sporadic	Current study
11_32014	USA: NY	Stool	2011 Oct 22	JEGX01.0004	W	178162	SRR618473	1/b	Sporadic	Current study
11_22186	USA: NY	Stool	2011 Jul 22	JEGX01.0004	W	178162	SRR618469	1/b	Sporadic	Current study
11_31312	USA: NY	Stool	2011 Oct 5	JEGX01.0004	W	178162	SRR618472	1/b	Sporadic	Current study
11_03844	USA: NY	Stool	2011 Feb 1	JEGX01.0004	W	178162	SRR618464	1/b	Sporadic	Current study
12_20343	USA: NY	Stool	2012 Jun 18	JEGX01.0004	W	178162	SRR652070	1/b	Sporadic	Current study
11_30508	USA: NY	Stool	2011 Oct 9	JEGX01.0004	W	178162	SRR618471	1/b	Sporadic	Current study
10_30147	Outside USA	Stool	2010 Aug 22	JEGX01.0004	W	178162	SRR610567	1/b	Sporadic	Current study
10_34599	USA: NY	Stool	2010 Sep 15	JEGX01.0004	W	178162	SRR611124	1/a	Sporadic	Current study
11_06235	USA: NY	Stool	2011 Feb 21	JEGX01.0004	W	178162	SRR618465	1/a	Sporadic	Current study
11_21079	USA: NY	Stool	2011 Jul 13	JEGX01.0004	W	178162	SRR618468	1/a	Sporadic	Current study
12_14700	USA: NY	Stool	2012 May 7	JEGX01.0004	B	178162	SRR651131	1/a	Sporadic	Current study
12_16076	USA: NY	Stool	2012 May 16	JEGX01.0004	B	178162	SRR651155	1/a	Sporadic	Current study
12_14693	USA: NY	Stool	2012 May 3	JEGX01.0004	B	178162	SRR651130	1/a	Sporadic	Current study
12_14703	USA: NY	Stool	2012 May 3	JEGX01.0004	B	178162	SRR651153	1/a	Sporadic	Current study
12_15721	USA: NY	Stool	2012 May 18	JEGX01.0021	B	178162	SRR651976	2/a	Sporadic	Current study
12_21567	USA: NY	Stool	2012 Jun 18	JEGX01.0021	B	178162	SRR652071	2/a	Sporadic	Current study
12_17892	USA: NY	?	2012 May 29	JEGX01.0021	B	178162	SRR651159	2/a	Sporadic	Current study
12_12288	USA: NY	Stool	2012 Apr 23	JEGX01.0021	B	178162	SRR651970	2	Sporadic	Current study
12_22891	USA: NY	Stool	2012 Jul 18	JEGX01.0021	B	178162	SRR652074	2	Sporadic	Current study
12_14487	USA: NY	Stool	2012 May 9	JEGX01.0021	B	178162	SRR651974	2/b	Sporadic	Current study
12_14699	USA: NY	Stool	2012 May 9	JEGX01.0021	B	178162	SRR653601	2/b	Sporadic	Current study
12_14089	USA: NY	Stool	2012 Apr 28	JEGX01.0021	B	178162	SRR651972	2/b	Sporadic	Current study
12_14982	USA: NY	Stool	2012 May 14	JEGX01.0021	B	178162	SRR653603	2	Sporadic	Current study
12_16414	USA: NY	Stool	2012 May 27	JEGX01.0021	B	178162	SRR651977	2/c	Sporadic	Current study
12_17211	USA: NY	Stool	2012 Jun 1	JEGX01.0021	B	178162	SRR651994	2/c	Sporadic	Current study
12_22983	USA: NY	Stool	2012 Jul 21	JEGX01.0021	B	178162	SRR652075	2/c	Sporadic	Current study
12_14697	USA: NY	Stool	2012 Apr 24	JEGX01.0021	B	178162	SRR653600	2/c	Sporadic	Current study
12_26681	USA: NY	Stool	2012 Aug 17	JEGX01.0021	B	178162	SRR652083	2/c	Sporadic	Current study
12_23418	USA: NY	Stool	2012 Jul 16	JEGX01.0021	B	178162	SRR652076	2/c	Sporadic	Current study
12_12016	USA: NY	Stool	2012 Apr 11	JEGX01.0021	B	178162	SRR651967	2/d	Sporadic	Current study
12_12205	USA: NY	Stool	2012 Apr 16	JEGX01.0021	B	178162	SRR651969	2/d	Sporadic	Current study
12_11922	USA: NY	Stool	2012 Apr 17	JEGX01.0021	B	178162	SRR651966	2/d	Sporadic	Current study
12_15432	USA: NY	Stool	2012 May 15	JEGX01.0021	B	178162	SRR651975	2/d	Sporadic	Current study
12_17893	USA: NY	Stool	2012 May 31	JEGX01.0021	B	178162	SRR651160	2/d	Sporadic	Current study
12_12071	USA: NY	Stool	2012 Apr 16	JEGX01.0021	B	178162	SRR651968	2/d	Sporadic	Current study
12_22120	USA: NY	Stool	2012 Jul 12	JEGX01.0021	B	178162	SRR652073	2/d	Sporadic	Current study
SL909	USA: NC	?	?	?	?	59999	SRR518849	2	Sporadic	(*1*)
10_35180	USA: NY	Stool	2010 Sep 12	JEGX01.0004	W	178162	SRR611128	3	LTCF	Current study
10_35417	USA: NY	Stool	2010 Sep 22	JEGX01.0004	W	178162	SRR611283	3	LTCF	Current study
10_34587	USA: NY	Stool	2012 Sep 20	JEGX01.0004	W	178162	SRR611123	3	LTCF	Current study
10_33369	USA: NY	Stool	2010 Sep 10	JEGX01.0004	W	178162	SRR610681	3	LTCF	Current study
10_35178	USA: NY	Stool	2010 Sep 13	JEGX01.0004	W	178162	SRR611126	3	LTCF	Current study
10_36119	?	?	2010 Sep 17	JEGX01.0004	W	178162	SRR611284	3	LTCF	Current study
10_34601	USA: NY	Stool	2010 Sep 13	JEGX01.0004	W	178162	SRR611125	3	LTCF	Current study
10_36979	USA: NY	Stool	2010 Oct 8	JEGX01.0004	W	178162	SRR618456	3	LTCF	Current study
10_38792	USA: NY	Stool	2010 Oct 29	JEGX01.0004	W	178162	SRR618459	3	LTCF	Current study
10_35183	USA: CT	Stool	2010 Sep 16	JEGX01.0004	W	178162	SRR611282	3	LTCF	Current study

Isolate tree label	Collection location	Isolation source	Collection date	PFGE pattern: first enzyme; second enzyme	NY MLVA	Bioproject	SRA accession no.	Clade/subclade†	Specific outbreak	Reference
10_35182	USA: NY	Stool	2010 Sep 12	JEGX01.0004	W	178162	SRR611281	3	LTCF	Current study
10_33371	USA: NY	Stool	2010 Sep 11	JEGX01.0004	W	178162	SRR610733	3	LTCF	Current study
10_33213	USA: NY	Stool	2010 Sep 10	JEGX01.0004	W	178162	SRR610680	3	LTCF	Current study
10_35181	USA: NY	Stool	2010 Sep 13	JEGX01.0004	W	178162	SRR611280	3	LTCF	Current study
10_36319	USA: NY	Stool	2010 Sep 28	JEGX01.0004	W	178162	SRR611285	3	LTCF	Current study
10_35179	USA: CT	Stool	2010 Sep 12	JEGX01.0004	W	178162	SRR611127	3	LTCF	Current study
622731–39	USA: IA	Environmental swab	2010 Aug 12	JEGX01.0004; JEGA26.0002	NA	52615	SRR518786	?	Sporadic	(*2*)
639016–6	USA: IA	Egg wash water	2010 Aug 19	JEGX01.0004; JEGA26.0002	NA	52617	SRR518813	?	Sporadic	(*2*)
648901 6–18	USA: OH	Environmental swab	2010 Sep 27	JEGX01.0004; JEGA26.0002	NA	62829	SRR518763	?	Sporadic	(*2*)
640631	USA: IA	Chicken feed-developer pullet	2010 Aug 17	JEGX01.0004; JEGA26.0002	NA	52619	SRR518800	?	Sporadic	(*2*)
648905 5–18	USA: OH	Environmental swab	2010 Oct 5	JEGX01.0004; JEGA26.0002	NA	62825	SRR518823	?	Sporadic	(*2*)
629164–37	USA: IA	Environmental swab	2010 Aug 30	JEGX01.0004; JEGA26.0030	NA	59539	SRR518757	?	Sporadic	(*2*)
485549–17	USA: IA	Environmental swab	2010 Aug 30	JEGX01.0004; JEGA26.0030	NA	59531	SRR518788	?	Sporadic	(*2*)
639672–50	USA: IA	Environmental swab	2010 Aug 31	JEGX01.0004; JEGA26.0002	NA	59543	SRR518818	?	Sporadic	(*2*)
77–0424	USA: AZ	Clinical	1977	JEGX01.0004; JEGA26.0002	NA	53259	SRR518840	?	Sporadic	(*2*)
77–1427	USA: RI	Clinical	1977	JEGX01.0004; JEGA26.0002	NA	60069	SRR518841	?	Sporadic	(*2*)
8b-1	USA: GA	?	?	NA	NA	60511	SRR518767	?	Sporadic	(*2*)
607307–6	USA :IA	Environmental swab	2010 Aug 16	JEGX01.0004; JEGA26.0031	NA	53263	SRR518859	?	Sporadic	(*2*)
50–3079	USA: NJ	Clinical	1950	NA; JEGA26.0002	NA	73685	SRR518824	?	Sporadic	(*2*)
2010K-1018	USA: NC	Clinical	2010 Apr 24	JEGX01.0004_JEGA26.0002	NA	52373	SRR518778	?	Sporadic	(*1*)
2010K-1010	USA: NC	Meringue	2010 Apr 28	JEGX01.0108_JEGA26.0002	NA	52375	SRR518808	?	Sporadic	(*1*)
12_18401	USA: NY	Stool	2012 Jun 15	JEGX01.0009	CR	178162	SRR651164	4	Beef	Current study
12_19824	USA: NY	Hamburger	2012 Jun 28	JEGX01.0009	CR	178162	SRR651168	4	Beef	Current study
12_18160	USA: NY	Stool	2012 Jun 11	JEGX01.0009	CR	178162	SRR651162	4	Beef	Current study
12_19798	USA: NY	Stool	2012 Jun 23	JEGX01.0009	CR	178162	SRR651166	4	Beef	Current study
12_18137	USA: NY	Stool	2012 Jun 12	JEGX01.0968	CR	178162	SRR651161	4	Beef	Current study
12_18526	USA: NY	?	2012 Jun 20	JEGX01.0009	CR	178162	SRR652067	4	Beef	Current study
12_18775	USA: NY	Stool	2012 Jun 15	JEGX01.0009	CR	178162	SRR652068	4	Beef	Current study
12_17486	USA: NY	Urine	2012 Jun 9	JEGX01.0009	CR	178162	SRR651158	4	Beef	Current study
12_18138	USA: NY	?	2012 Jun 18	JEGX01.0009	CR	178162	SRR651184	4	Beef	Current study
12_21314	USA: NY	Stool	2012 Jun 28	JEG01.0843	CR	178162	SRR651175	4	Beef	Current study
SE10	USA: ME	Chicken ovary	?	JEGX01.0004_JEGA26.0002	NA	41919	SRR518774	?	Sporadic	(*1*)
CVM 56–3991	USA: TN	Clinical	1956	JEGX01.0004_JEGA26.0002	NA	59991	SRR518789	?	Sporadic	(*1*)
SE15–1	USA: ME	Poultry environment	?	JEGX01.0004_JEGA26.0002	NA	59987	SRR518785	?	Sporadic	(*1*)
SE8a	USA: GA	?	?	?	NA	41915	SRR518853	?	Sporadic	(*1*)
18569	Mexico	Poultry	?	JEGX01.0002_NA	NA	41929	SRR518826	?	Sporadic	?
SE30663	USA: MD	Ground turkey	?	JEGX01.0019_JEGA26.0010	NA	42905	SRR518835	?	Sporadic	?
10_33603	USA: NY	Stool	2010 Sep 14	JEGX01.0004	B	178162	SRR618450	?	Sporadic	Current study

Isolate tree label	Collection location	Isolation source	Collection date	PFGE pattern: first enzyme; second enzyme	NY MLVA	Bioproject	SRA accession no.	Clade/subclade†	Specific outbreak	Reference
12_21313	USA: NY	?	2012 Jul 5	JEGX01.0004	B	178162	SRR651174	?	Sporadic	Current study
12_14895	USA: NY	Stool	2012 May 15	JEGX01.0004	B	178162	SRR651154	?	Sporadic	Current study
12_16086	USA: NY	Stool	2012 May 25	JEGX01.0004	B	178162	SRR651156	?	Sporadic	Current study
12_26778	USA: NY	Stool	2012 Aug 17	JEGX01.0004	B	178162	SRR652084	?	Sporadic	Current study
2010K-1455	USA: PA	Clinical	2010 May 26	JEGX01.0004_JEGA26.0002	NA	52509	SRR518782	?	Sporadic	(*1*)
CDC 2010K-1457	USA: PA	Clinical	2010 May 27	JEGX01.0004_JEGA26.0002	NA	52383	SRR518855	?	Sporadic	(*1*)
22510–1	USA: NC	Chicken	?	JEGX01.0004_JEGA26.0002		60075	SRR518784	?	Sporadic	(*1*)
12_16608	USA: NY	Stool	2012 May 29	JEGX01.0004	B	178162	SRR651993	?	Sporadic	Current study
10_35184	USA: NY	Stool	2010 Sep 16	JEGX01.0004	AE	178162	SRR618454	?	Sporadic	Current study
10_34213	USA: NY	Stool	2010 Sep 13	JEGX01.0004	B	178162	SRR618451	?	Sporadic	Current study
12_24729	USA: NY	Stool	2012 Jul 31	JEGX01.0004	B	178162	SRR652080	?	Sporadic	Current study
12_19760	USA: NY	?	2012 Jun 19	JEGX01.0004	W	178162	SRR652069	?	Sporadic	Current study
12_23426	USA: NY	Stool	2012 Jul 18	JEGX01.0034	B	178162	SRR652077	?	Sporadic	Current study
10_31528	USA: NY	Stool	2010 Aug 26	JEGX01.0004	W	178162	SRR610679	?	Sporadic	Current study
77–2659	USA: SD	Clinical	1977	JEGX01.0004; JEGA26.0002	NA	60071	SRR518843	?	Sporadic	(*2*)
SL913	USA: NC	?	?	?	NA	60001	SRR518848	?	Sporadic	(*1*)
78–1757	USA: NE	Clinical	1978	JEGX01.0004; JEGA26.0002	NA	60073	SRR518811	?	Sporadic	(*2*)
13183–1	USA: IA	Chicken breast	?	JEGX01.0004_JEGA26.0002	NA	59995	SRR518812	?	Sporadic	(*1*)

*PFGE, pulsed-field gel electrophoresis; NY MLVA, New York multilocus variable-number tandem-repeat analysis; SRA; sequence read archive; USA, United States; TN, Tennessee; NA, not available (NY-MLVA type was not determined); MN, Minnesota; CA, California; IA, Iowa; ?, unknown; NC, North Carolina; LCTF, isolates assigned to long-term care facility outbreak; CT, Connecticut; OH, Ohio; AZ, Arizona; RI, Rhode Island; GA, Georgia; NJ, New Jersey; ME, Maine; MD, Maryland; PA, Pennsylvania; SD, South Dakota; NE, Nebraska.
†Clade (number) and subclade (letter) designation used in Figure 2. Isolates without clade designation did not harbor clustered isolates that were sequenced in this study.

References

1. Köser CU, Holden MT, Ellington MJ, Cartwright EJ, Brown NM, Ogilvy-Stuart AL, et al. Rapid whole-genome sequencing for investigation of a neonatal MRSA outbreak. N Engl J Med. 2012;366:2267–75. PubMed http://dx.doi.org/10.1056/NEJMoa1109910

2. Lienau EK, Strain E, Wang C, Zheng J, Ottesen AR, Keys CE, et al. Identification of a salmonellosis outbreak by means of molecular sequencing. N Engl J Med. 2011;364:981–2. PubMed http://dx.doi.org/10.1056/NEJMc1100443
